# Single-Nuclei RNA Sequencing Data Analysis: Cardiomyocyte-Specific Modified-mNA Targeting Cyclin D2 Turns Subpopulations of Adult Pig Cardiomyocytes, Fibroblasts, and Smooth Muscle Cells Back to Neonatal Characteristics at Day 2 After SMRT Intervention

**DOI:** 10.3390/biom16101477

**Published:** 2026-10-10

**Authors:** Thanh Nguyen, Kaili Hao, Daniel J. Garry, Jianyi Zhang

**Affiliations:** 1 Department of Biomedical Engineering, School of Medicine and School of Engineering, University of Alabama at Birmingham, Birmingham, AL 35294, USA; khao@uab.edu (K.H.); jayzhang@uab.edu (J.Z.); 2 Department of Medicine, School of Medicine, University of Minnesota, Minneapolis, MN 55455, USA; garry@umn.edu; 3 Department of Medicine, Division of Cardiovascular Disease, School of Medicine, University of Alabama at Birmingham, Birmingham, AL 35294, USA

**Keywords:** CCND2, modified RNA, CM-SMRTs, cardiomyocyte, heart regeneration

## Abstract

Naturally, pig hearts cannot recover from ischemic reperfusion injury; however, pig hearts receiving cardiomyocyte-specific modified-RNA (CM-SMRTs) targeting Cyclin D2 (*^CCND2^*CM-SMRTs) demonstrated significantly better cardiac function, compared to the untreated hearts receiving Green Fluorescent Protein-induced modified RNA (*^GFP^*CM-SMRTs). In this report, we generated and analyzed single-nuclei RNA sequencing (snRNAseq) data to examine the mechanism by which *^CCND2^*CM-SMRTs regulate the cardiomyocyte cell cycle and improve cardiac function in the whole heart. The snRNAseq data from *^CCND2^*CM-SMRTs-treated pig hearts (~2-month-old) were integrated with the data from age-matched *^GFP^*CM-SMRTs-treated hearts, harvested on day 2 and day 28 after treatment, as well as with naïve neonatal day 1 and 2 (with regenerative potential) and with naïve 2-month-old (without regenerative potential) data. SnRNAseq data from all cardiac cell types were analyzed using an Autoencoder pipeline. Six cardiomyocytes (CM1–CM6), four fibroblasts (FB1–FB4), six smooth muscle cells (SMC1–SMC4), five endothelial cells (EC1–EC5), four macrophages (Mph1–Mph4), and two T-cell (Tcell1 and Tcell2) clusters were defined. While CCND2 is well-known as a major cell cycle driver from G1 to S phase, the snRNAseq data did not indicate any significant changes regarding cell cycle activity among *^CCND2^*CM-SMRTs-treated pigs, compared to *^GFP^*CM-SMRTs pigs, on day 2. Rather, clusters CM1, FB1, and SMC1 were observed in neonatal pig hearts and at the injury-border zone, where CM-SMRTs were delivered, in *^CCND2^*CM-SMRT-treated hearts on day 2 after treatment; these clusters did not appear in any remote zones, which were not subjected to ischemic reperfusion and CM-SMRTs effect, or in any untreated and naïve hearts. However, on day 28, clusters of FB1, and SMC1 were no longer observed, and only CM1 were prominently seen in border zone of *^CCND2^*CM-SMRT-treated hearts. Intercellular communication analysis revealed that CM1 may impact other non-cardiomyocyte clusters early after SMRT intervention via the BMP and PDGF signaling pathways. Thus, the analysis suggested that *^CCND2^*CM-SMRTs may drive a subset of pig cardiac cells toward a neonatal state, in which pig hearts still retain regenerative capacity.

## 1. Introduction

Heart failure remains a leading cause of mortality worldwide [1]. In heart failure, the heart muscle loses its contractility, especially after a large amount of cardiomyocyte death following ischemic injuries [2]. Mammalian cardiomyocytes (CMs) exit the cell cycle shortly after birth [3] and, thus, cannot naturally proliferate in response to injury in adult mammals. In addition, studies using regenerative heart models [3,4,5,6,7,8,9,10] suggested that it was possible to reactivate cardiomyocyte proliferation in adult mammals by overexpressing cell cycle regulators [11,12,13]. Among the cell cycle regulators, Cyclin D2 [*CCND2*] was critical in the G1/S transition during the cell cycle process [14,15,16,17]. In vitro, overexpressing CCND2 increased cell proliferation in human-induced pluripotent stem cell-derived cardiomyocytes [18]. In vivo, adult pigs treated with cardiomyocyte-specific Cyclin D2-overexpressed modified RNA (*^CCND2^*CM-SMRTs) demonstrated robust cardiomyocyte proliferation, which was the key driver in remuscularizing the heart following ischemic reperfusion injury [19]. Meanwhile, hearts receiving cardiomyocyte-specific Green Fluorescent Protein-induced modified RNA (*^GFP^*CM-SMRTs) did not activate cardiomyocyte proliferation; thus, *^GFP^*CM-SMRTs hearts did not recover after ischemic reperfusion [19]. However, the mechanism by which *^CCND2^*CM-SMRTs impact cardiomyocytes has not been thoroughly studied; this question will be examined by generating and analyzing single-nuclei RNA sequencing data from pigs receiving *^CCND2^*CM-SMRTs, compared with pigs receiving *^GFP^*CM-SMRTs, after ischemic reperfusion injury. We will examine two possible answers regarding this mechanism: (1) by direct cell cycle-specific analysis using Seurat cell cycle scoring [20,21] and cell cycle-specific Autoencoding [6]; and (2) by possibly driving the heart toward the neonatal state. Because neonatal pig hearts still maintain regenerative capacity on neonatal day 1 and 2 [5,10], we hypothesized that *^CCND2^*CM-SMRTs may drive the adult pig cardiac cells, especially cardiomyocytes, toward neonatal characteristics.

## 2. Materials and Methods

### 2.1. Animals

All experimental protocols were approved by The Institutional Animal Care and Use Committee (IACUC, # IACUC-20216, Approved in 2016, revised 1 December 2024) of the University of Alabama, Birmingham, and performed in accordance with the National Institutes of Health Guide for the Care and Use of Laboratory Animals (NIH publication No 85–23). Pigs were purchased from Prestage Farm, Inc. (West Point, MS, USA) and cared for as described previously [19].

### 2.2. Tissue and Nuclei Isolation

Frozen pig hearts preserved from our previous *^CCND2^*CM-SMRTs study [19] were reused in this study. Protocols for handling the tissue were approved by the Institutional Animal Care and Use Committee (IACUC) of the University of Alabama, Birmingham, and were performed in accordance with the National Institutes of Health Guide for the Care and Use of Laboratory Animals (NIH publication No. 85–23). Briefly, ischemic reperfusion was induced in ~2-month-old pigs, and *^CCND2^*CM-SMRTs or *^GFP^*CM-SMRTs (also called nGFP in our previous study) were directly delivered into the ischemic area via intramyocardial injection before closing the chest. This protocol was applied in our previous works [19]. The following pig groups were reused in this study (day 0: inducing ischemic reperfusion and CM-SMRTs treatment):

− Pig receiving *^GFP^*CM-SMRTs and harvested on day 2 (GFP-D2,), 4 samples, 2 male, 2 female. According to previous studies [19,22], the target genes induced by CM-SMRTs reached peak expression between days 2 and 7 after delivery.
− Pig receiving *^GFP^*CM-SMRTs and harvested on day 28 (GFP-D28), 4 samples, all female.
− Pig receiving *^CCND2^*CM-SMRTs and harvested on day 2 (CCND2-D28), 4 samples, all female.
− Pig receiving *^CCND2^*CM-SMRTs and harvested on day 28 (CCND2-D28), 4 samples, 2 male, 2 female.

Nuclei isolation was done as in our previous works [9,23]. Briefly, tissues at both the ischemic-border zone (BZ, nearby the apex) and remote (RZ, >2.5 cm above the apex) were cut while submerged in cold phosphate-buffered saline (PBS) or UW solution, washed to remove blood, transferred into 1 mL lysis buffer (10 mM Tris-HCl, 10 mM NaCl, 3 mM MgCl_2_, 0.1% Nonidet™ P40 Substitute, and 50 U/mL RNase inhibitor in DEPC-treated water), cut into smaller pieces, and aspirated with the lysis buffer into a 50 mL tube; then, 10 mL of lysis buffer was added, the tissues were ground for 20–30 s, and more lysis buffer was added. The mixture was placed on ice for 10 min, filtered with 100 μm and 70 μm strainers, and centrifuged for 5 min at 700 relative centrifugal force (rcf) and 4 °C; then, the supernatant was removed, and the pellet was resuspended in 10 mL nuclei wash and resuspension buffer (1× PBS, 1.0% bovine serum albumin [BSA], and 50 U/mL RNase inhibitor). The suspension was passed through a 40 μm strainer, and the nuclei were centrifuged again for 5 min at 700 rcf and 4 °C; then, the supernatant was removed, the pellet was resuspended in 1 mL nuclei wash and resuspension buffer, and the nuclei were centrifuged a third time for 5 min at 700 rcf and 4 °C. The supernatant was removed; then, the pellet was resuspended in 5 mL sucrose cushion buffer I (2.7 mL Nuclei PURE 2 M Sucrose Cushion Solution and 300 μL Nuclei PURE Sucrose Cushion Buffer, Sigma-Aldrich, St. Louis, MO, USA) and mixed with 10 mL sucrose buffer. The mixture was layered over 5 mL of sucrose cushion buffer in a second Eppendorf tube and then centrifuged for 60 min at 13,000 *g* and 4 °C. All but 100 μL of the supernatant was removed, the nuclei were resuspended in nuclei wash and resuspension buffer, and the solution was passed through a 40 μm strainer; then, the nuclei concentration was determined with a cell counter or hemocytometer and adjusted to 1000 nuclei/μL. The nuclei were placed on ice, stained with propidium iodide for 5 min, and then immediately processed via the 10× Genomics^®^ Single-Cell Protocol.

### 2.3. Processing of snRNAseq Data

snRNAseq data were processed as described previously [5,6,9,23]. Sample demultiplexing, barcode processing, and gene counting were performed with Cell Ranger Single-Cell Software v.3.10 (https://www.10xgenomics.com/support/software/cell-ranger/9.0/release-notes/cr-release-notes-archive, accessed on 23 September 2026). Reads were aligned to the Sscrofa11.1 pre-mRNA reference genome [24]. Only confidently mapped reads with valid barcodes were used to generate the gene-barcode matrix. DoubletFinder [25] was applied to filter out doublets.

Data from all samples, including the previously acquired data from naïve CTL-P1, -P2 (4 samples, 1 male, 3 female), and -P56 pigs (3 samples, 1 male, 2 female) [9,23], were integrated and normalized via the Seurat R package [26], reduced to 10 dimensions with an Autoencoder [8], and visualized via Uniform Manifold Approximation (UMAP) [27]. Clustering was performed with the UMAP density-based clustering (dbscan) algorithm [28]. Then, the cardiac cell types were identified as in the Autoencoder pipeline [8]. The snRNAseq data of each cell type (cardiomyocyte, fibroblast, endothelial cell, smooth muscle cell, and immune cell) were processed by the second-round Autoencoders; then, cell clusters were identified.

The marker statistics for each cluster and the gene ontology enrichment analysis were performed as in our previous works [5]. Differentially expressed genes were defined according to three criteria: (1) statistical significance (Wilcoxon rank-sum test; *p* < 0.01 for CSA, *p* < 10^–6^ for CSF analysis) [29]; (2) expression in at least 10% of cells within a cluster; and (3) ≥1.5-fold higher mean expression compared with all other clusters. Genes meeting these criteria were functionally annotated using the DAVID tool [30]. Enrichment analysis was limited to manually curated Gene Ontology [31] and KEGG [32], databases. To correct for multiple hypothesis testing, enrichment was considered significant if *p* < 0.01 or Benjamini-adjusted *p* < 0.05 [33]. Enrichment score for each pathway in each cluster was reported as the base-10 logarithm of the Benjamini-adjusted *p*-value; the enrichment score is balanced between the score among upregulated genes (positive) and downregulated genes (negative). For each cell type, trajectory analysis was completed by the Monocle toolkit [34]. Then, CellChat toolkit [35] was applied to analyze the ligand–receptor intercellular communication among cardiomyocyte, fibroblast, endothelial cell, smooth muscle cell, and immune cell clusters.

In addition, cardiomyocyte snRNA-seq data were also analyzed by cell cycle-specific methods to identify whether a proliferative cardiomyocyte subpopulation would emerge. Two independent methods were applied. First, cell cycle scoring within the Seurat toolkit [26] was applied. The cell cycle scoring method has been widely applied in quantifying the enrichment of cells at the G1, S, and G2/M phases in many studies. Second, we used cell cycle-specific Autoencoder, which has been applied to detect proliferative cardiomyocytes and endothelial cells from fetal, neonatal, and regenerative mouse and pig hearts [5,6,36,37]. The cell cycle-specific Autoencoder aims to detect cell subpopulations in which all five proliferation markers AURKB, MKI67, INCENP, BIRC5, and CDCA8 are co-localized.

## 3. Results

### 3.1. Five Cell Types Were Identified from snRNAseq Data from Pigs Receiving ^CCND2^CM-SMRTs After Ischemic Reperfusion

There were 219,783 nuclei that were isolated from both injury-border zone (BZ, nearby the apex) and remote zone (RZ, >2 cm above the apex) of *^CCND2^*CM-SMRT and *^GFP^*CM-SMRT hearts after ischemic reperfusion. The hearts were explanted on day 2 (D2—active CM-SMRTs) and day 28 (D28—CM-SMRTs no longer active) after treatment. The snRNAseq data from these 219,783 nuclei were integrated with snRNAseq data from naïve neonatal days 1 and 2 (CTL-P1+2), which still retained regenerative capacity [38], and from naïve 2-month-old (CTL-P56) pig hearts, in which the regenerative capacity had been lost [5,9,23]. After doublet removal [25], the integrated snRNAseq data comprised 254,029 nuclei, in which 209,275 were from the injured pigs.

**Figure 1:**
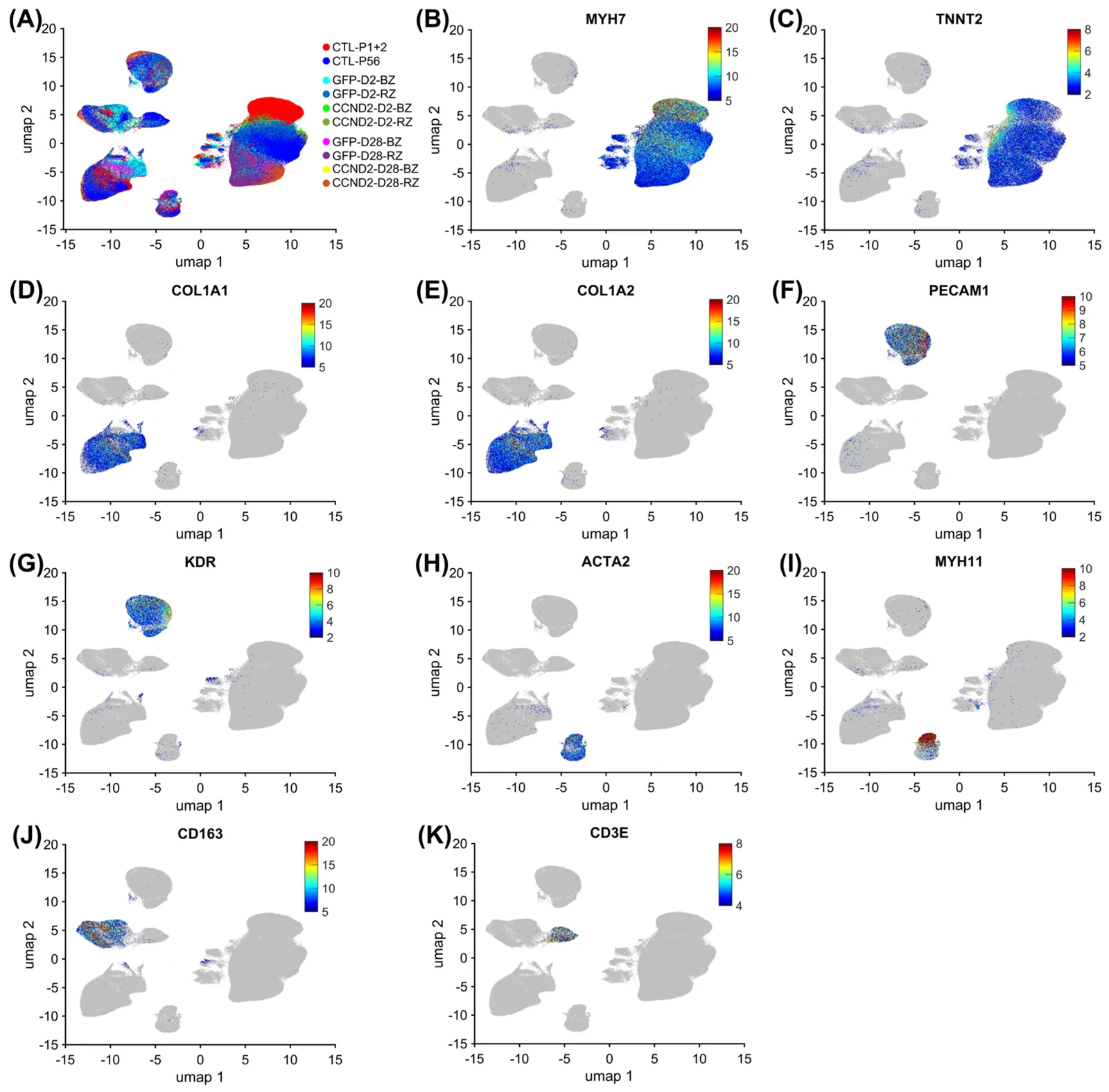
Identification of cardiac cell type from snRNAseq data of *^CCND2^*CM-SMRTs, *^GFP^*CM-SMRTs hearts after ischemic reperfusion, integrated with data from naïve hearts. (**A**) Distribution of cells in different groups and timepoints is displayed in the 2D umap. (**B**–**K**) Distributions of cells enriching cell type-specific markers: cardiomyocytes MYH7 (**B**) and TNNT2 (**C**), cardiac fibroblast COL1A1 (**D**) and COL1A2 (**E**), endothelia cell PECAM1 (**F**) and KDR (**G**), smooth muscle cell ACTA2 (**H**) and MYH11 (**I**), macrophage CD163 (**J**), and T cell CD3E (**K**); gray cells in each panel did not highly express the corresponding markers.

After being processed by Seurat [21] and Autoencoder [8] toolkits, five major clusters of cells emerged (Figure 1). Cells from all animal groups were well distributed across the five clusters, indicating minimal batch effects after Autoencoder processing. Cell-type identities for each cluster were determined based on the established markers: cardiomyocytes, by Troponin T2 Cardiac Type [TNNT2], and Myosin Heavy Chain [MYH7] (Figure 1B,C); fibroblasts, by Collagen Type I Alpha 1 Chain [COL1A1] and Collagen Type I Alpha 2 Chain [COL1A2] (Figure 1D,E); endothelial cells, by Platelet And Endothelial Cell Adhesion Molecule 1 [PECAM1] and Kinase Insert Domain Receptor [KDR] (Figure 1F,G); smooth muscle cells, by Actin Alpha 2 Smooth Muscle [ACTA2] and Smooth Muscle Myosin Heavy Chain 11 [MYH11]) (Figure 1H,I); and immune cells, Macrophage-Associated Antigen [CD163] and CD3 Epsilon Subunit Of T-Cell Receptor Complex [CD3E] (Figure 1J,K). Metadata, including sample group, cell barcode, and cell type, for each cell are in Supplementary Table S1.

### 3.2. A Large Proportion of Cardiomyocytes with ^CCND2^CM-SMRTs Treatment Demonstrated the Neonatal P1&2 Transcriptomic Profile

**Figure 2:**
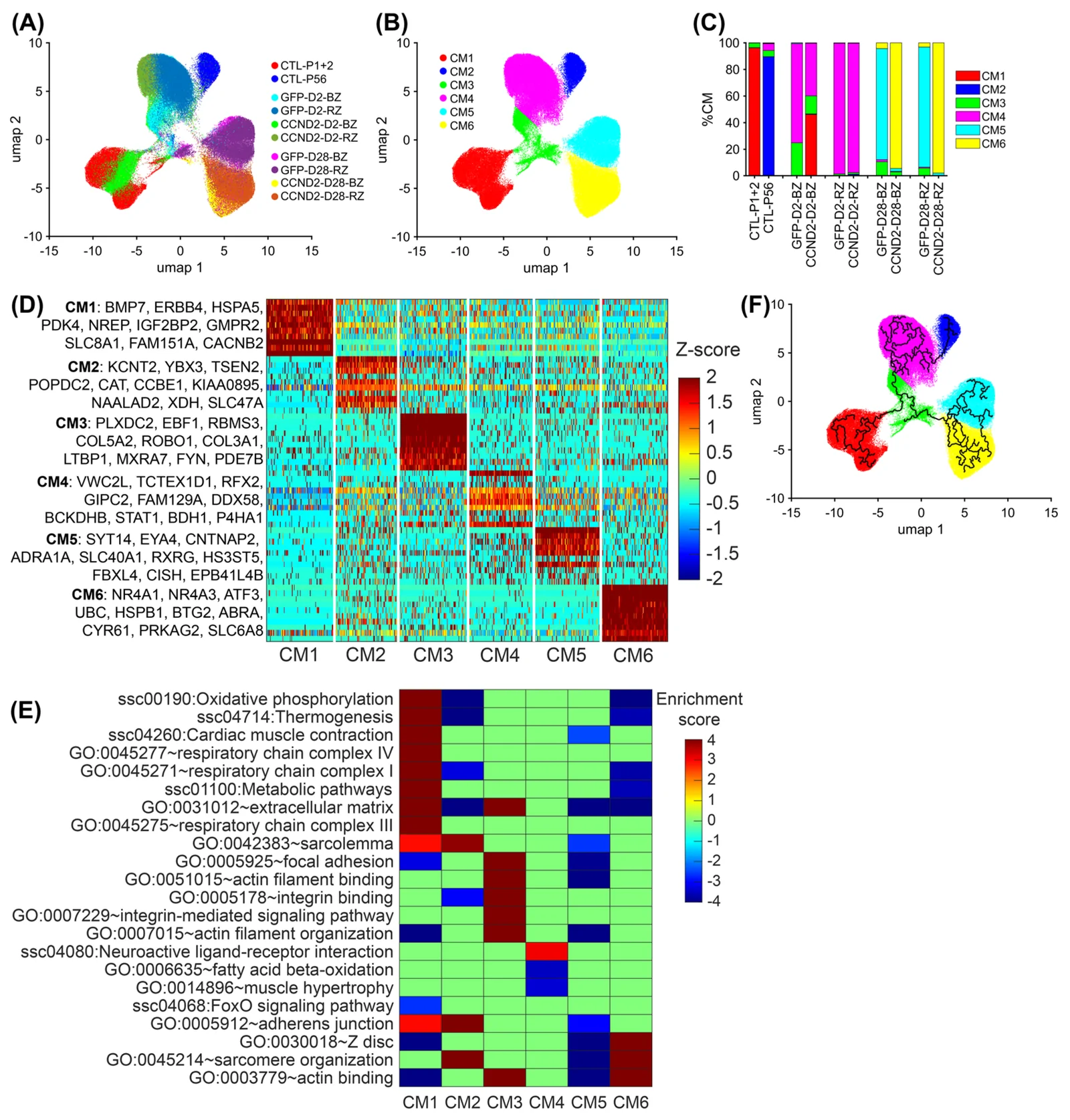
Analysis of cardiomyocyte snRNAseq data. (**A**) Distribution of cardiomyocytes in different groups and timepoints is displayed in the 2D umap. (**B**) Location and boundary of six cardiomyocyte clusters (CM1–CM6) on the umap. (**C**) Proportions of each cardiomyocyte cluster in each heart group are displayed in a stacked bar chart. (**D**) The expression of the top 10 genes, sorted by fold-change, is displayed within each cluster as a heat map; each column represents a randomly selected cell. (**E**) Enrichment scores of selected gene ontologies and signaling pathways in each cluster. (**F**) Trajectory analysis among all cardiomyocyte clusters by Monocle toolkit; the black line indicates possible transitions among these clusters.

In Figure 2A, cardiomyocyte snRNAseq data (145,417 nuclei) were processed by another Autoencoder, and displayed by the Uniform Manifold Approximation and Projection (UMAP) [27] toolkit. Consistent with our previous analyses using the same naïve heart samples [4,9,23], the cardiomyocytes of CTL-P1+2 hearts were well separated from those of CTL-P56 hearts (Figure 2A). The majority of *^CCND2^*CM-SMRT- and *^GFP^*CM-SMRT-treated cardiomyocytes on treatment day 2 (D2), at both BZ and RZ, were more aligned with CTL-P56, consistent with the age of the pigs when ischemic reperfusion and CM-SMRT treatments were performed (~2 months). Meanwhile, the majority of *^CCND2^*CM-SMRT- and *^GFP^*CM-SMRT-treated cardiomyocytes on D28 were separated from others, and a separation between *^CCND2^*CM-SMRTs and *^GFP^*CM-SMRTs could be observed.

Therefore, six cardiomyocyte clusters (CM1–CM6) were defined (Figure 2B). Among them, cluster CM1 consisted of more than 95% cardiomyocytes in CTL-P1+2 hearts. Cluster CM1 did not appear in CTL-P56 and *^GFP^*CM-SMRT-treated hearts at any timepoints. However, in *^CCND2^*CM-SSMRTs-treated hearts CM1 appeared only in the border zone at treatment D2, the timepoint and location at which CM-SMRTs were effective (~40% cardiomyocytes) (Figure 2C). Cluster CM1 encompassed a number of positive cardiomyocyte cell cycle regulators, including Bone Morphogenetic Protein 7 [BMP7] [39], Erb-B2 Receptor Tyrosine Kinase 4 [ERBB4] [40], and Heat Shock Protein Family A (Hsp70) Member 5 [HSPA5] [5] (Figure 2D). Also, CM1 upregulated metabolic pathways, such as oxidative phosphorylation and respiratory chain complex (Figure 2E, Supplementary Table S2); this result was consistent with our previous reports on CTL-P1 cardiomyocyte transcriptomic analysis [23].

Cluster CM2 exclusively appeared in the naïve CTL-P56 hearts (Figure 2C) and comprised more than 90% CTL-P56 cardiomyocytes. The sarcolemma, sarcomere organization, and adherens-junction pathways were highly enriched in CM2 (Figure 2E), indicating that CM2 represented mature cardiomyocytes. Cluster CM3 was observed only in hearts that underwent ischemic reperfusion, with either *^CCND2^*CM-SMRTs or *^GFP^*CM-SMRTs treatment, primarily in the border zone. Also, CM3 proportions were the highest at the border zone of day 2 after ischemic reperfusion (*^GFP^*CM-SMRTs: 24.58%, *^CCND2^*CM-SMRTs: 13.54%), then declined sharply on day 28 (*^GFP^*CM-SMRTs: 10.39%, *^CCND2^*CM-SMRTs: 2.84%) (Figure 2C). In addition, the integrin-related pathways, which were well-known in cardiomyocyte responses after injury [41], were upregulated in CM3 (Figure 2E). Therefore, CM3 likely indicated immediate cardiomyocyte responses to myocardial damage following ischemic reperfusion. Meanwhile, cluster CM4, which is neighbor to CM2, was observed in all pigs on day 2 after ischemic reperfusion (~2 months old). Furthermore, CM4 proportions were higher at the remote zones (Figure 2C) of both *^CCND2^*CM-SMRT- and *^GFP^*CM-SMRT-treated hearts. Thus, CM4 likely represented cardiomyocytes that did not respond to ischemic reperfusion or were not transfected with *^CCND2^*CM-SMRTs.

In addition, clusters CM5 and CM6 only appeared in hearts on day 28 after ischemic reperfusion and treatments (Figure 2C). CM5 was exclusive to and comprised more than 90% of *^GFP^*CM-SMRT cardiomyocytes in both border and remote zones. Meanwhile, CM6 was exclusive to, and comprised more than 90% of *^CCND2^*CM-SMRT cardiomyocytes in both border and remote zones. The Z-disk, sarcomere organization, and actin-binding pathways, which were required for cardiomyocyte contractile performance, were upregulated in CM6; however, these pathways were downregulated in CM5, consistent with our observation in cardiomyocyte snRNAseq data of heart failure patients [42]. Altogether, CM5 and CM6 indicated different outcomes in cardiomyocytes at the whole heart, even though only border-zone cardiomyocytes demonstrated an immediate response following ischemic reperfusion, and CM-SMRTs were only transfected into border-zone cardiomyocytes.

Surprisingly, the cell cycle-specific analyses did *NOT* indicate any major differences between *^CCND2^*CM-SMRTs and *^GFP^*CM-SMRT cardiomyocytes. Seurat cell cycle scoring indicated that the enrichment (proportion) of G1, S, and G2/M cardiomyocytes were relatively similar among all heart groups (Supplementary Figure S1A). Also, among six cardiomyocyte clusters, none of them had an explicitly higher proportion of G2/M stages (Supplementary Figure S1B). Furthermore, the cell cycle-specific Autoencoder did *NOT* show any area where all proliferation markers AURKB, MKI67, INCENP, BIRC5, and CDCA8 co-enriched (Supplementary Figure S2Ai–Av). Still, the cell cycle-specific Autoencoder separated CTL1-+2 and CTL-P56 cardiomyocytes and showed that only *^CCND2^*CM-SMRTs-D2-BZ overlapped with neonatal CTL-P1+2 cardiomyocytes (Supplementary Figure S2B–E). Then, from the trajectory analysis (Figure 2F), cluster CM3 appeared to be an intermediate state between adult-heart cardiomyocyte (clusters CM2 and CM4), neonatal-heart cardiomyocyte (cluster CM1), and post-I/R cardiomyocytes (cluster CM5 and CM6). The trajectory at CM3 could either go to CM1 or CM5 and CM6. Surprisingly, CM3 presented in both *^CCND2^*CM-SMRTs- and *^GFP^*CM-SMRTs-treated hearts. Altogether, these results suggest that *^CCND2^*CM-SMRTs more likely induces CM proliferation by shifting post-I/R cardiomyocytes at the CM3-switch toward the neonatal stage, rather than by directly changing cardiomyocyte cell cycle phases.

### 3.3. A Proportion of Fibroblasts with ^CCND2^CM-SMRTs Treatment Demonstrated the Neonatal P1&2 Transcriptomic Profile

**Figure 3:**
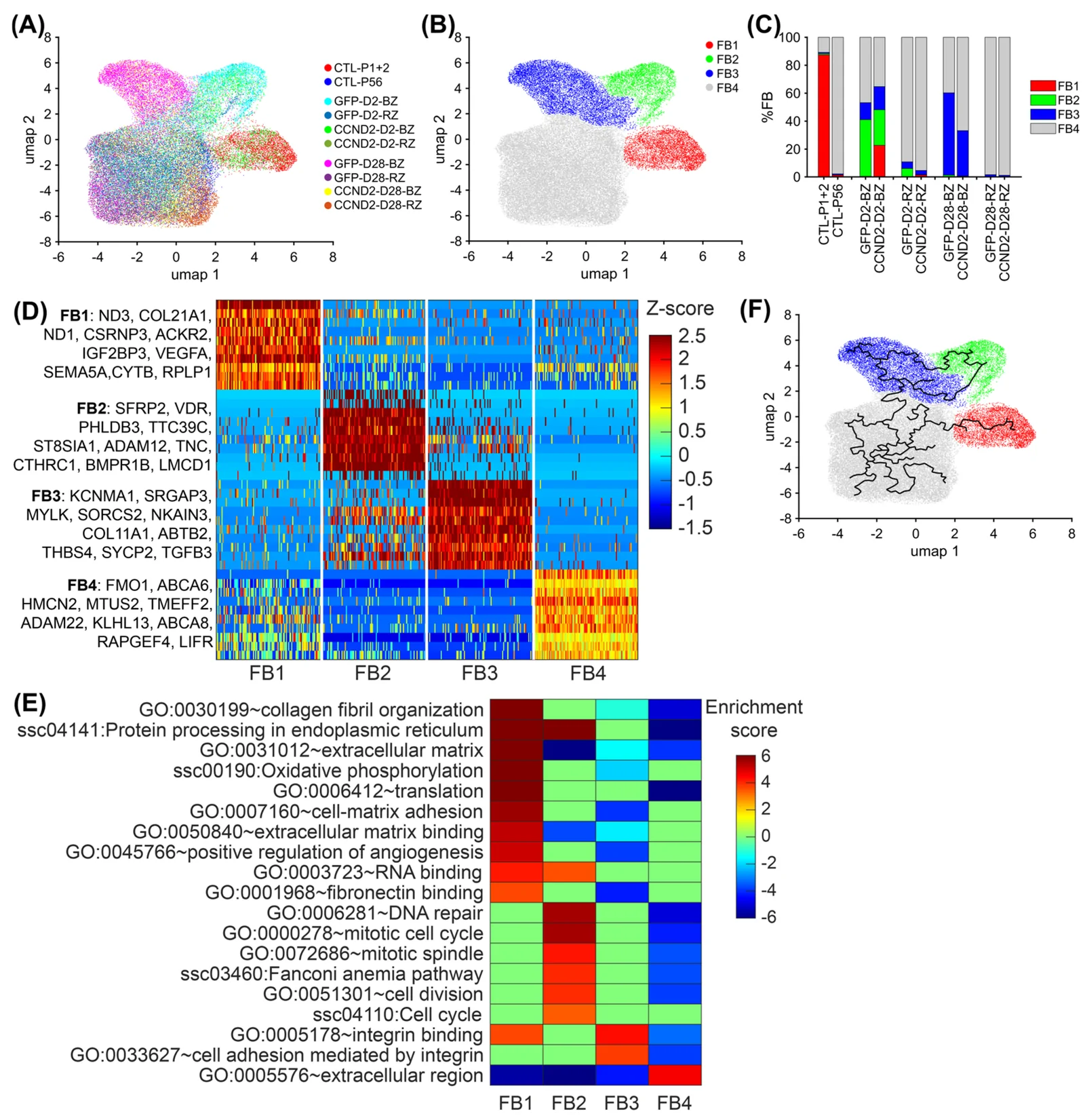
Analysis of fibroblast snRNAseq data. (**A**) Distribution of fibroblasts in different groups and timepoints is displayed in the 2D umap. (**B**) Location and boundary of four fibroblast clusters (FB1–FB4) on the umap. (**C**) Proportions of each fibroblast cluster in each heart group were displayed in a stacked bar chart. (**D**) The expression of the top 10 genes, sorted by fold-change, is displayed as a heat map within each cluster; each column represents a randomly selected cell. (**E**) Enrichment scores of selected gene ontologies and signaling pathways in each cluster. (**F**) Trajectory analysis among all fibroblast clusters by Monocle toolkit; the black line indicates possible transitions among these clusters.

In Figure 3A, fibroblast snRNAseq data (48,632 cells) were processed by another Autoencoder and UMAP [27] toolkits. Moderate separation among fibroblasts of CTL-P1+2, border-zone of ischemic reperfusion on day 2 and day 28 (both *^CCND2^*CM-SMRTs and *^GFP^*CM-SMRTs treatments) was observed. Thus, four fibroblast clusters (FB1–FB4) were defined (Figure 3B).

Cluster FB1 consisted of ~90% fibroblasts in CTL-P1+2 hearts. Collagen-family genes, such as COL21A1, collagen-related, extracellular matrix, and positive regulation of angiogenesis (such as VEGFA [43]) pathways, were upregulated in FB1 (Figure 3D,E, Supplementary Table S3). These results were consistent with the distinct fibroblast and extracellular matrix characteristics of neonatal hearts in other studies [44]. Interestingly, FB1 also comprised ~20% of the fibroblasts in the border zone of hearts treated with *^CCND2^*CM-SMRTs on day 2 after treatment, and it did not appear in any other heart groups.

In addition, cluster FB2 primarily appeared at the border zone of hearts on day 2 after ischemic reperfusion, including both *^CCND2^*CM-SMRTs and *^GFP^*CM-SMRTs treatments (Figure 3C). The DNA repair, mitotic cell cycle, cell division, and Fanconi anemia pathways were upregulated in FB2 (Figure 3E), suggesting that FB2 represents proliferative fibroblasts. The proportion of FB2 in *^GFP^*CM-SMRTs hearts (40.58%) was significantly higher than in *^CCND2^*CM-SMRTs hearts (25.61%), which was consistent with the reduction in fibrosis observed in *^CCND2^*CM-SMRTs treatment [19]. Cluster FB3 only appeared at the border zone in both *^CCND2^*CM-SMRTs- and *^GFP^*CM-SMRTs-treated hearts. Meanwhile, cluster FB4 comprised more than 90% of fibroblasts in naïve CTL-P56 and remote zones of *^CCND2^*CM-SMRTs- and *^GFP^*CM-SMRTs-treated hearts.

Trajectory analysis (Figure 3F) indicated that cluster FB1 was directly linked to cluster FB4 and was not linked to any other fibroblast clusters. Cluster FB3 appeared to be the intermediate stage between FB4 and the proliferative FB3. Because FB4 is the primary (>95%) fibroblast cluster in adult P56 and the remote zone of I/R pigs, FB1 in *^CCND2^*CM-SMRTs hearts was unlikely to be from new-proliferative fibroblasts; rather, it is more likely from the ‘resident’ fibroblasts after I/R and under *^CCND2^*CM-SMRTs treatments.

### 3.4. A Small Proportion of Smooth Muscle Cells with ^CCND2^CM-SMRTs Treatment Demonstrated the Neonatal P1&2 Transcriptomic Profile

**Figure 4:**
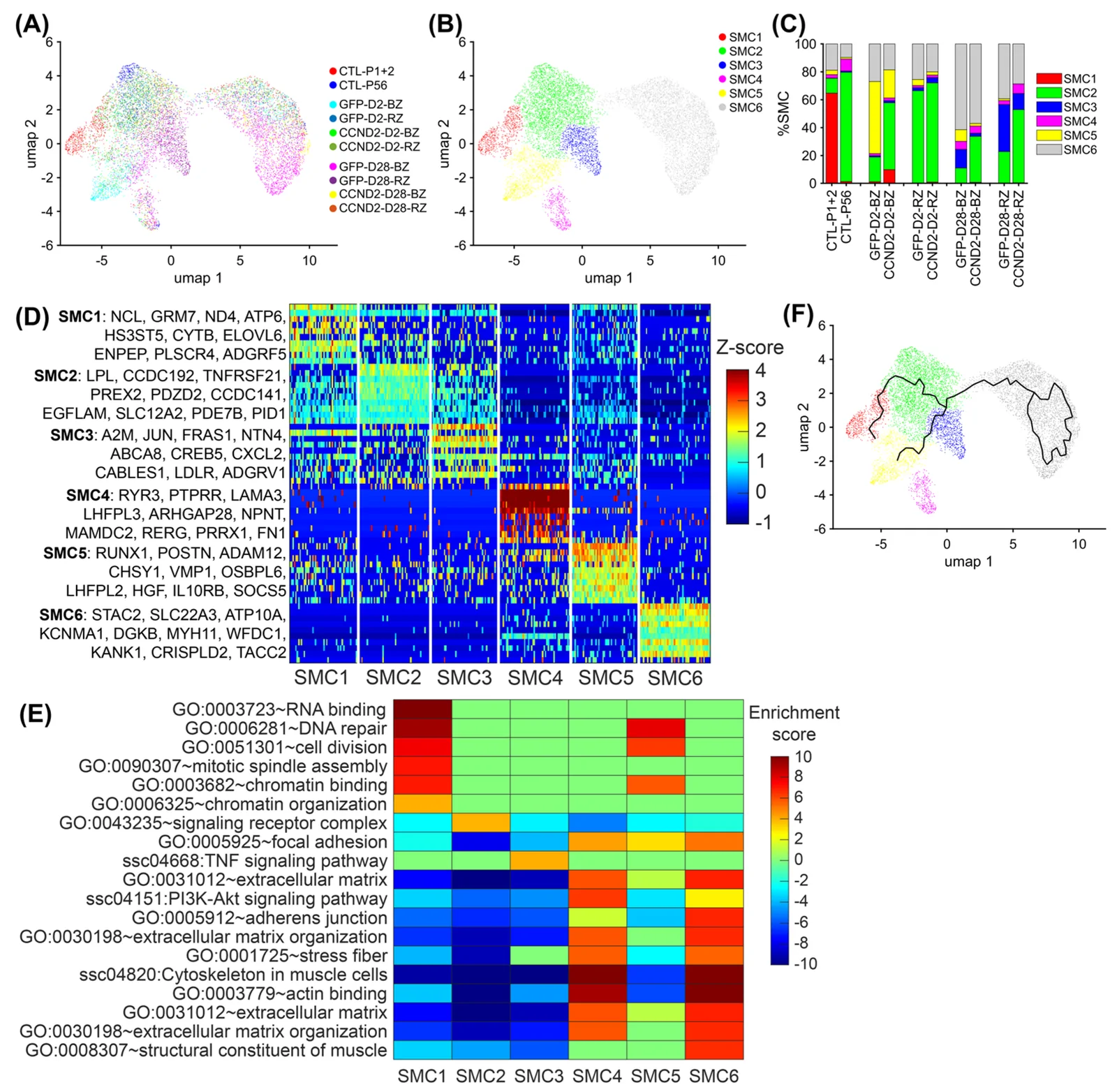
Analysis of smooth muscle cell snRNAseq data. (**A**) Distribution of smooth muscle cells in different groups and timepoints is displayed in the 2D umap. (**B**) Location and boundary of six smooth muscle cell clusters (SMC1–SMC6) on the umap. (**C**) Proportions of each smooth muscle cell cluster in each heart group were displayed in a stacked bar chart. (**D**) The expression of the top 10 genes, sorted by fold-change, is displayed within each cluster as a heat map; each column represents a randomly selected cell. (**E**) Enrichment scores of selected gene ontologies and signaling pathways in each cluster. (**F**) Trajectory analysis among all smooth muscle cell clusters by Monocle toolkit; the black line indicates possible transitions among these clusters.

In Figure 4A, smooth muscle cell snRNAseq data (9764 cells) were processed by another Autoencoder and UMAP [27] toolkits. Moderate separation among smooth muscle cells of CTL-P1+2, the border-zone of ischemic reperfusion on day 2, between both *^CCND2^*CM-SMRTs and *^GFP^*CM-SMRTs treatments was observed. Thus, six smooth muscle cell clusters (SMC1–SMC6) were defined (Figure 4B).

Cluster SMC1 consisted of ~60% smooth muscle cells in CTL-P1+2 hearts and ~10% smooth muscle cells at the border zone in *^CCND2^*CM-SMRTs-treated hearts on day 2 (Figure 4C); otherwise, SMC1 did not appear in any other groups. The cell division, DNA repair, and chromatin binding pathways were upregulated in SMC1 (Figure 4D,E, Supplementary Table S4), as well as in SMC5, which primarily appeared at the border zone of both *^CCND2^*CM-SMRTs-treated and *^GFP^*CM-SMRTs-treated hearts. This result indicated that SMC1 and SMC5 were likely proliferative, which was consistent with previous reports that the coronary vasculature undergoes a period of rapid growth and remodeling shortly after birth [45], as well as increased angiogenesis following injury in both regenerative and non-regenerative hearts [36]. Trajectory analysis (Figure 4F) indicates that both SMC1 and SMC5 were likely from cluster SMC2.

In addition, SMC2 was the major (78.56%) smooth muscle cell cluster in CTL-P56 and remote zones of *^CCND2^*CM-SMRTs- and *^GFP^*CM-SMRTs-treated hearts (Figure 4C). On day 28 after ischemic reperfusion, the proportion of SMC2 in *^GFP^*CM-SMRTs-treated hearts declined to 22.72%; meanwhile, SMC2 remained the major smooth muscle cell cluster in *^CCND2^*CM-SMRT-treated hearts on both day 2 and day 28 after ischemic reperfusion. This result indicated that *^CCND2^*CM-SMRTs treatment may have protective effects on smooth muscle cells. Meanwhile, cluster SMC3 was highly enriched at both border and remote zones of *^GFP^*CM-SMRTs-treated hearts (Figure 4C); here, the Tumor Necrosis Factor (TNF) signaling pathway, which was associated with inflammation and oxidative stress [46], was upregulated (Figure 4E). In addition, we did not observe significant differences in the proportions of SMC4 and SMC6 between the *^CCND2^*CM-SMRTs and *^GFP^*CM-SMRTs groups.

### 3.5. Endothelial Cell Heterogeneity Following ^CCND2^CM-SMRTs and ^GFP^CM-SMRTs Treatments

**Figure 5:**
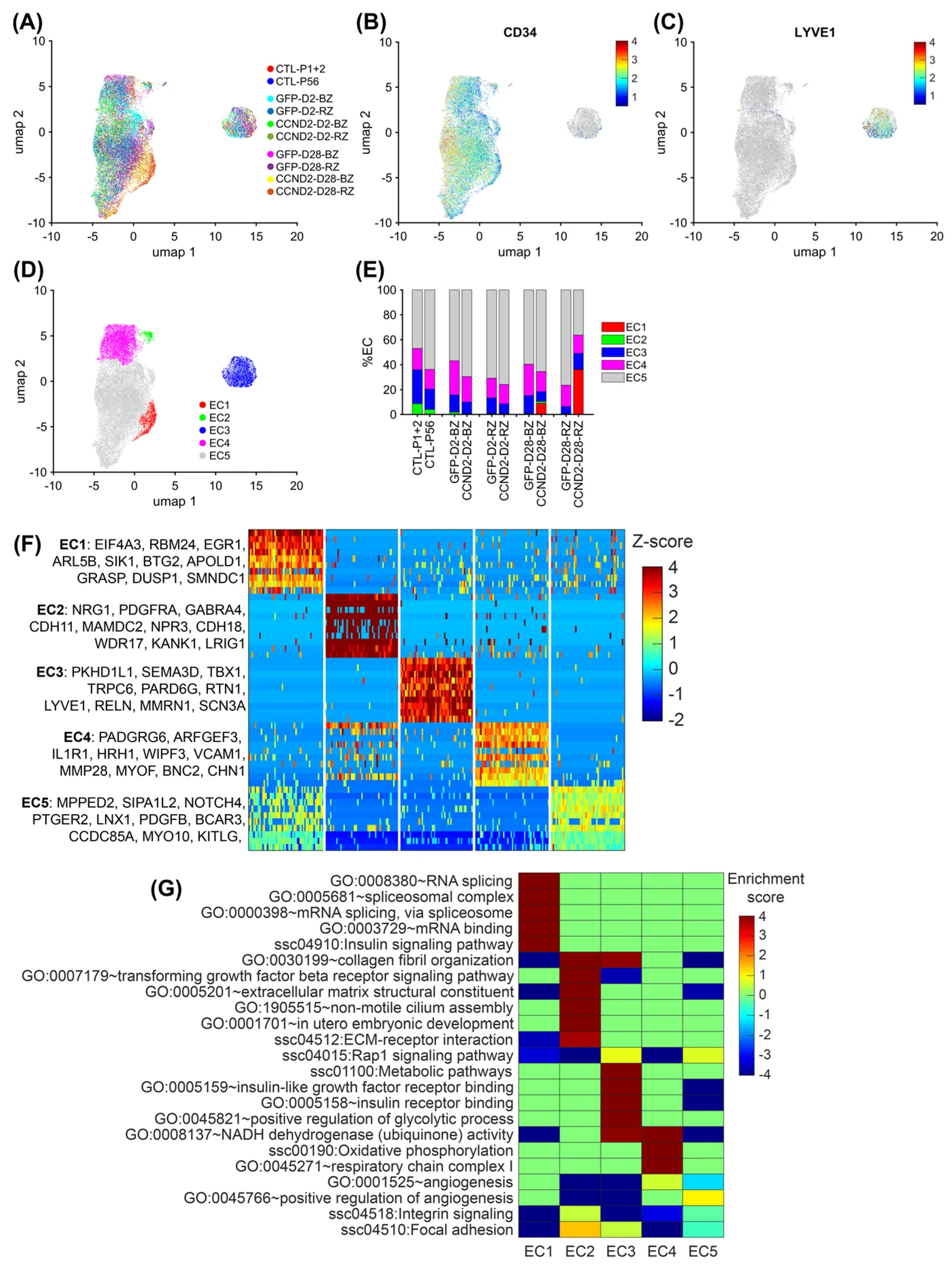
Analysis of endothelial cell snRNAseq data. (**A**) Distribution of endothelial cells in different groups and timepoints is displayed in the 2D umap. (**B**,**C**) Distributions of cells expressing vascular endothelial cell marker CD34 (**B**) and lymphatic endothelial cell marker LYVE1 (**C**); gray cells in each panel did not highly express the corresponding markers. (**D**) Location and boundary of six smooth muscle cell clusters (SMC1–SMC6) on the umap. (**E**) Proportions of each fibroblast cluster in each heart group were displayed in a stacked bar chart. (**F**) The expression of the top 10 genes, sorted by fold-change, within each cluster is displayed as a heat map; each column represents a randomly selected cell. (**G**) Enrichment scores of selected gene ontologies and signaling pathways in each cluster.

In Figure 5A, endothelial cell snRNAseq data (26,446 cells) were further processed by Autoencoder and UMAP [27] toolkits. By the landscape of cells expressing vascular endothelial cell (CD34 Molecule [47]—Figure 5B) and lymphatic endothelial cell (Lymphatic Vessel Endothelial Hyaluronan Receptor 1 [LYVE1] [48]—Figure 5C), five endothelial cell clusters (EC1–EC5) were defined (Figure 5D); among them, EC3 was the only lymphatic endothelial cell cluster. Cluster EC1 only appeared in *^CCND2^*CM-SMRT-treated hearts on day 28 (border zone: 9.00%, remote zone: 36.03%—Figure 5E); here, RNA splicing factors, such as Eukaryotic Translation Initiation Factor 4A3 [EIF4A3] and RNA Binding Motif Protein 24 [RBM24], were upregulated (Figure 5F,G, Supplementary Table S5). Cluster EC2 primarily appeared in CTL-P1+2 pigs (Figure 5E); here, Endo-mesenchymal transition marker Platelet-Derived Growth Factor Receptor Alpha [PDGFRA], the TGFβ signaling, and collagen fibril organization pathways were upregulated (Figure 5F,G). Thus, EC2 demonstrated the subpopulation of endothelial cells with Endo-mesenchymal transition, and consistent with our previous reports of endothelial cells in regenerative pig heart [36]. Otherwise, cluster EC4 was enriched in metabolic pathways, such as oxidative phosphorylation and respiratory chain complex I, and EC5 was the major endothelial cell cluster across all heart groups. The trajectory among endothelial cell clusters was presented in Supplementary Figure S3A.

### 3.6. Immune Cell Heterogeneity Following CCND2CM-SMRTs and GFPCM-SMRTs Treatments

**Figure 6:**
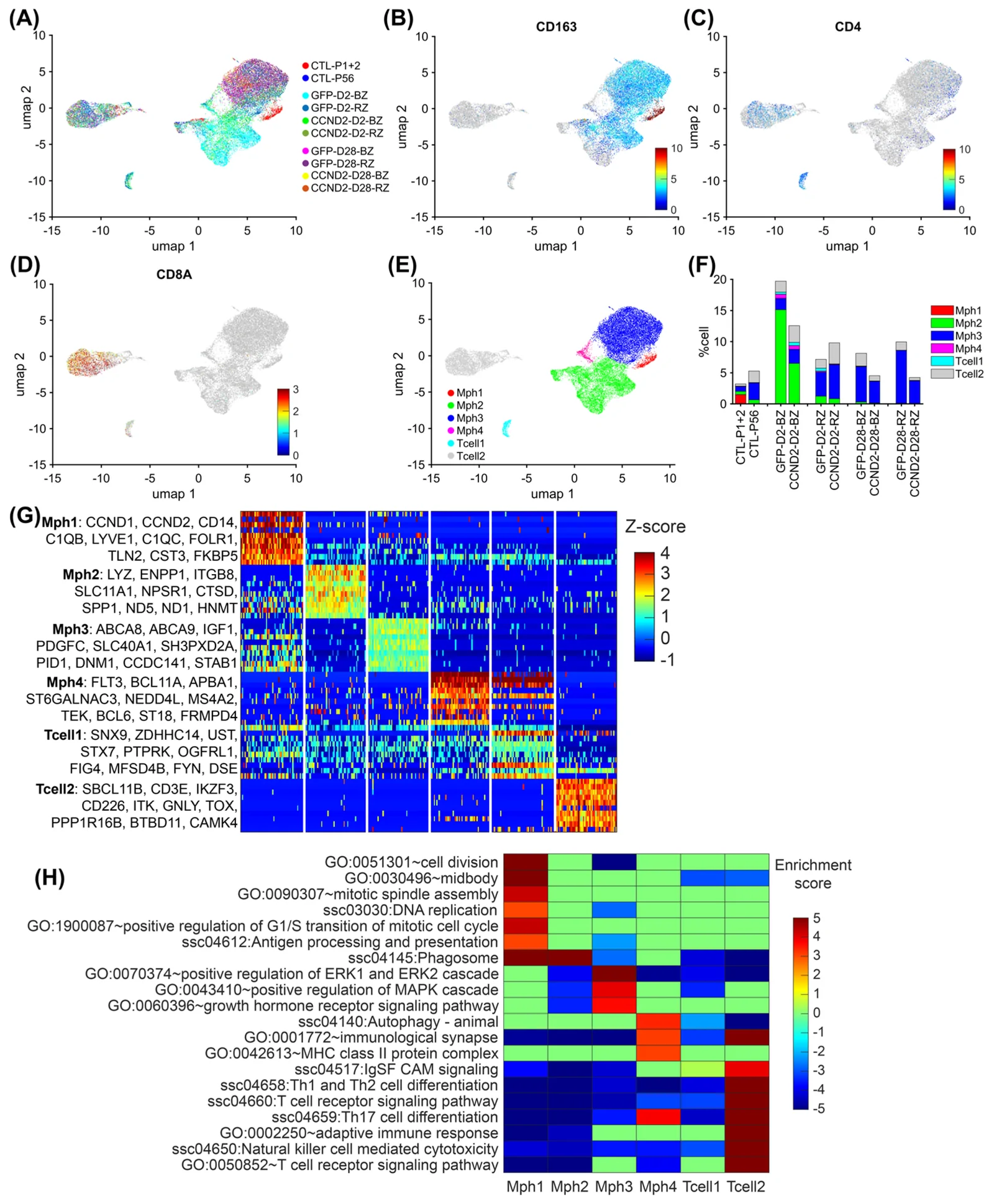
Analysis of immune cell snRNAseq data. (**A**,**C**–**G**) (**A**) Distribution of immune cells in different groups and timepoints is displayed in the 2D umap. (**B**,**D**) Distributions of cells expressing macrophage marker CD163 (**B**), and T cell marker CD4 (**C**) and CD8A (**D**); gray cells in each panel did not highly express the corresponding markers. (**E**) Location and boundary of six smooth muscle cell clusters (SMC1–SMC6) on the umap. (**F**) Proportions of each fibroblast cluster in each heart group were displayed in a stacked bar chart. (**G**) The expression of the top 10 genes, sorted by fold-change, within each cluster is displayed as a heat map; each column represents a randomly selected cell. (**H**) Enrichment scores of selected gene ontologies and signaling pathways in each cluster.

In Figure 6A, immune cell snRNAseq data (23,770 cells) were further processed by Autoencoder and UMAP [27] toolkits. By the landscape of cells expressing macrophage (CD163—Figure 6B) and T-cell (CD4 and CD8A—Figure 6C,D) markers, four macrophage clusters (Mph1–Mph4) and two T-cell clusters (Tcell1 and Tcell2) were defined (Figure 6E). Interestingly, the total percentage of immune cells in *^CCND2^*CM-SMRT-treated hearts was lower than in *^GFP^*CM-SMRTs-treated hearts (Figure 6F). Cluster Mph1 only appeared in CTL-P1+2 hearts (Figure 6F); here, cell division, DNA replication, and positive regulation of G1/S transition of mitotic cell cycle pathways (i.e., CCND1 and CCND2) were upregulated (Figure 6G,H, Supplementary Table S6). Thus, Mph1 was likely the proliferative macrophage, which was consistent with the regenerative macrophage phenotype in regenerative hearts [49]. Mph2 was presented only at the border zone in pigs on day 2 after ischemic reperfusion (Figure 6F); here, the phagosome pathway, which indicated the initial responses of macrophages to debris [50], was upregulated (Figure 6H).

In addition, the T-cell cluster appears in all heart groups (Figure 6F). Among them, the T cell 1 (Tcell1) proportion was relatively small; also, the T cell receptor signaling, Th1, Th2, and Th17 pathways were downregulated in Tcell1, indicating that Tcell1 might not be a fully functional T cell. The majority of T-cells were in cluster Tcell2. Tcell2 was detected in all heart groups; however, the Tcell2 proportion in *^CCND2^*CM-SMRT-treated hearts was lower than in *^GFP^*CM-SMRTs-treated hearts The trajectory among immune cell clusters was presented in Supplementary Figure S3B.

### 3.7. Intercellular Communication Analysis Suggested That CM1 Likely Interacted with Other Non-CM Cell Clusters via the BMP and PDGF Signaling

**Figure 7:**
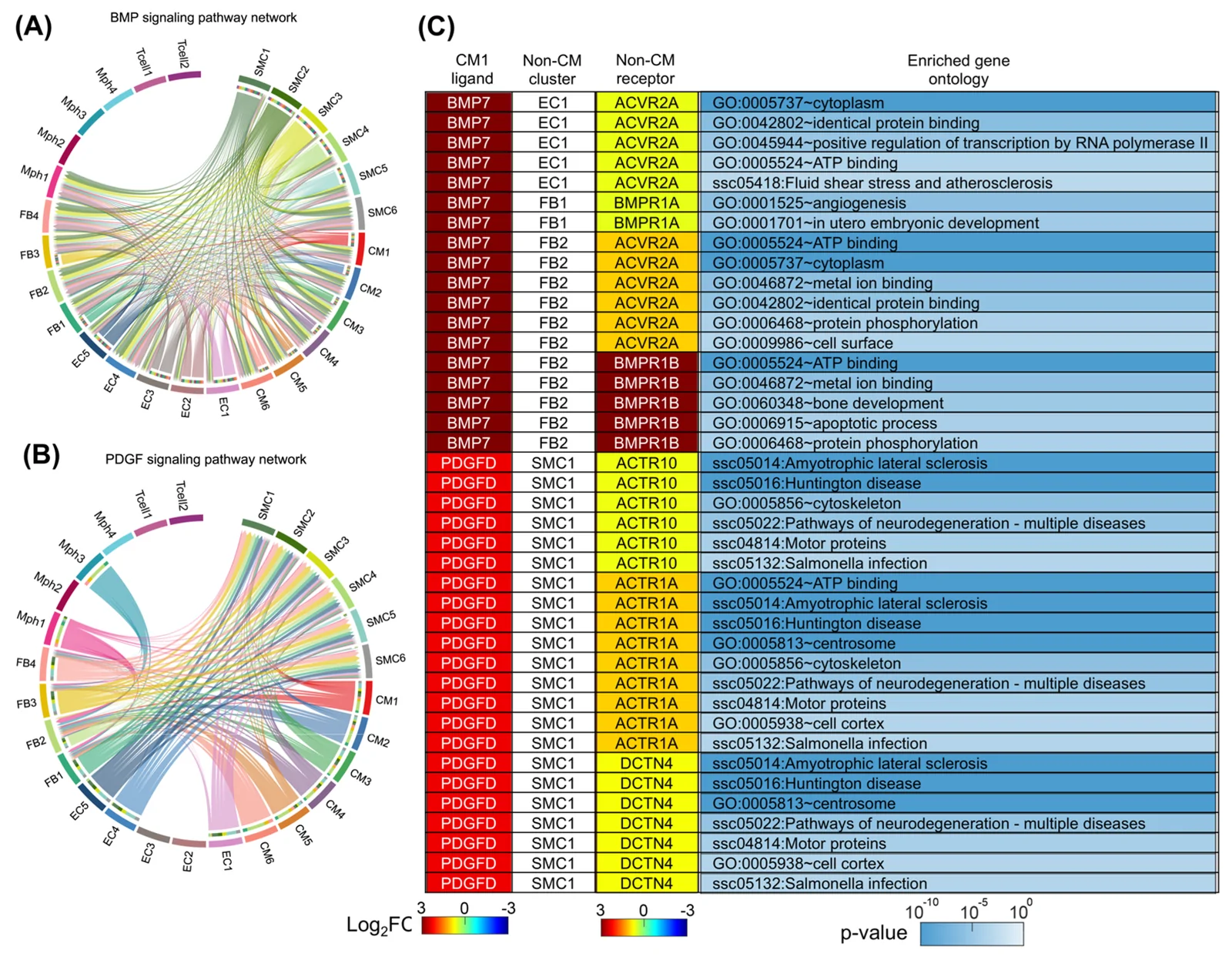
Intercellular communication between CM1 and other cell clusters. (**A**,**B**) Chord diagram (by Monocle toolkit) demonstrating the BMP (**A**) and PDGF (**B**) signaling pathways connecting multiple cell clusters, especially from CM1. (**C**) Table showing the CM1 ligand–non-CM receptor–downstream enriched pathways triplet; fold-changes in the CM1 ligand and non-CM receptor were color-coded with the yellow-red heatmap; enrichment *p*-value for each signaling pathway was color-coded with the white-blue heatmap.

The analysis of cardiomyocytes, fibroblasts, and smooth muscle cells indicated that clusters CM1, FB1, and SMC1 appeared in both neonatal and *^CCND2^*CM-SMRTs-D2 hearts; furthermore, these clusters likely indicated the responses to *^CCND2^*CM-SMRTs treatment rather than to the new/proliferative subpopulations. Also, cluster EC1 was exclusive to *^CCND2^*CM-SMRTs hearts, even though this cluster did not appear in the neonatal hearts. Because *^CCND2^*CM-SMRTs only turned on CCND2 in cardiomyocytes, which resulted in the appearance of CM1 on day 2 after treatment, the changes in non-cardiomyocytes were most likely due to intercellular communication from CM1 to other cell types. Then, intercellular communication analysis by Cellchat toolkit [35] indicated that CM1 communication was primarily happening via the BMP and PDGF signaling pathways (Figure 7A,B, Supplementary Table S7), which are well-known for their roles in cardiac development and maturity [51,52,53,54,55,56,57,58]. Specifically, ligands *BMP7* and *PDGFD* were highly upregulated in CM1 (Figure 7C). BMP7 interacted with receptors ACVR2A (upregulated in EC1 and FB2) and BMPR1A (upregulated in FB1). Meanwhile, PDGFD interacted with receptors ACTR10, ACTR1A, and DCTN4, which were upregulated in cluster SMC1 (Figure 7C). Downstream of these ligand–receptor interactions, 40 downstream signaling pathways and gene ontologies were enriched (Figure 7C), such as the in utero embryonic development pathway (downstream of BMP7-BMPR1A) in cluster FB1.

## 4. Discussion

CCND2, as well as other members of the Cyclin D family, is known for cell cycle progression from the G1 to the S phase in multiple cancer cell lines [59,60]. In vitro experiments using human-induced pluripotent stem cell-derived cardiomyocyte (hiPSC-CM) showed that overexpressing CCND2 increased hiPSC-CM proliferation [18]. Therefore, CCND2 was chosen as the target for the CM-SMRTs therapy in our previous work [19], with the expectation that CCND2 would directly ‘turn on’ cell cycle activity in cardiomyocytes. However, the cell cycle-specific analysis outcomes in Supplementary Figure S1, in which there was no significant shift from G1 to S in cardiomyocytes in the *^CCND2^*CM-SMRT group, as well as no cluster enriching all proliferation markers (AURKB, MKI67, INCENP, BIRC5, and CDCA8), came as a surprise. Although our previous study [19], as well as this study, did not contradict the fact that CCND2 promoted cardiomyocyte proliferation, this study suggested that the intermediate mechanism by which CCND2 promotes cardiomyocyte proliferation was not likely enhancing the G1-to-S progression.

A concise viewpoint of how adult-heart cardiomyocytes can regain proliferative capacity is ‘turning back the clock’: the cells become less mature and have neonatal or embryonic cardiomyocyte characteristics [61,62,63,64,65,66]. The snRNAseq data of *^CCND2^*CM-SMRT cardiomyocytes, which overlapped with neonatal P1 and P2 cells in cluster CM1, were consistent with this viewpoint. Also, the FOXO signaling [67], focal adhesion [68], Z-disk [69], and actin-binding pathways [70], which are well-known signaling pathways in mature cardiomyocytes, were downregulated in CM1; these results further indicated the immaturity of CM1. Also, some distinct phenotypic characteristics of less mature cardiomyocytes are a smaller cross-sectional area and a higher fraction of mononucleated cells; these characteristics were also observed in our previous *^CCND2^*CM-SMRT study [19]. This study was among the first sets of evidence for the association between CCND2 and cardiomyocyte maturity, which has not been well-studied. Nevertheless, the immature state of cardiomyocytes in *^CCND2^*CM-SMRT treatment appeared to be only within a short period.

In addition, it is important to note that cardiomyocyte proliferation and immaturity (toward fetal and neonatal states) may not be always correlated. In our previous works, the pig heart, after neonatal-day-1 apical resection, regenerated the whole myocardium for the second time from a day-28 myocardial infarction without any treatment; the snRNAseq and other proliferative assays confirmed the reactivation of proliferative cardiomyocytes in the regenerative hearts, but snRNAseq indicated no overlap between the regenerative and neonatal/fetal groups [4,6,23]. Meanwhile, this study, along with reports of other gene therapies inducing cardiomyocyte proliferation [12,62,63,71,72,73], suggested less-matured cardiomyocytes were associated with increased proliferation.

In addition to cardiomyocytes, overlaps between *^CCND2^*CM-SMRTs and neonatal cells were also observed in smooth muscle cells (cluster SMC1) and fibroblasts (cluster FB1). Intercellular communication analysis suggested that cardiomyocytes with CCND2 overexpression, which appeared in the CM1 cluster, could drive fibroblasts and smooth muscle cells toward a more neonatal phenotype via the BMP and PDGF ligand–receptor mechanisms. Furthermore, the BMP and PDGF signaling may be related to the communication of other cell subpopulations, instead of just CM1. However, how CCND2 activated BMP and PDGF ligands in cardiomyocytes was still unknown.

The following limitations may still occur in this study. First, *^CCND2^*CM-SMRTs can be transfected into non-cardiomyocyte (off-target); and while the design of CM-SMRTs guarantees cardiomyocyte specificity [74], it may take more than two days after transfection to achieve this effect [22]. Second, we have not examined how the BMP and PDGF signaling may induce the neonatal smooth muscle and fibroblast cluster (FB1 and SMC1) in *^CCND2^*CM-SMRT hearts on day 2 after treatment; investigating these reasons required knowledge of how mature heart fibroblasts and smooth muscle cells could turn ‘immature’ via CCND2 or other pathways related to CCND2. These questions are yet to be investigated. Third, we have not investigated how temporarily immature cardiomyocytes and non-cardiomyocytes could turn to maturity on day 28 after *^CCND2^*CM-SMRT transfection, particularly due to the lack of samples at intermediate timepoints between day 2 and day 28. Forth, although the pigs’ sex did not seem to heavily influence the main analytic results, the pig samples in the *^CCND2^*CM-SMRTs-D2 and *^GFP^*CM-SMRTs-D28 were all female, due to the availability of pigs at the time of surgery. And fifth, all pigs received carprofen for pain relief on the same day after ischemic reperfusion in this study, as well as in our other studies of endogenous heart regeneration [9,10,38]. While it is unlikely that carprofen impacts myocardial regeneration and cardiac fibrosis, carprofen is known for its anti-inflammatory effect [75], and therefore could be a co-factor in the analysis of immune cells.

## 5. Conclusions

In conclusion, the snRNAseq analysis of *^CCND2^*CM-SMRT-treated hearts indicated that the mechanism of *^CCND2^*CM-SMRTs involved the maturity of the heart, including cardiomyocytes and non-cardiomyocytes.

## Data Availability

The snRNASeq data of *^CCND2^*CM-SMRT-treated and *^GFP^*CM-SMRT-treated hearts was deposited into the Gene Expression Omnibus database, accession number GSE348334 (https://www.ncbi.nlm.nih.gov/geo/query/acc.cgi?acc=GSE348334, accessed on 23 September 2026). The analytic results (in Matlab version 2024b) were available at https://uab.box.com/s/yr6v6xdlczn2bna5hf3oujh8iph8m0xy, accessed on 23 September 2026.

## Supplementary Materials

The following supporting information can be downloaded at: https://www.mdpi.com/article/10.3390/biom16101477/s1. Figure S1. Seurat’s Cell-cycle scoring analysis of cardiomyocyte. (A) Stacked bar plot showed the distribution of cardiomyocytes at the G1, S, and G2M phase; each column corresponds to a sample group. (B) The same stacked bar plot to (A), but each column corresponds to a cardiomyocyte cluster. Figure S2. Cell-cycle-specific Autoencoder analysis of cardiomyocyte. (A) Distribution of cells expressing five proliferation markers *AURKB* (Ai), *MKI67* (Aii), *INCENP* (Aiii), *BIRC5* (Aiv), and *CDCA8* (Av); each dot represent a cell, the dot color reflect the expression of each marker, and gray cell did not express the marker; we did not detect any area in which all markers were enriched. (B–E) Distribution of cardiomyocyte in specific heart groups, in pair: (B) neonatal P1+2 (Bi) vs. adult P56 (Bii); (C) CCND2-D2-BZ (Ci) vs. GFP-D2-BZ (Cii); (D) CCND2-D2-RZ (Di) vs. GFP-D2-RZ (Dii); (E) CCND2-D28-BZ (Ei) vs. GFP-D28-BZ (Eii); (F) CCND2-D28-RZ (Fi) vs. GFP-D28-RZ (Fii). Figure S3. Trajectory analysis of endothelial cells (A) and immune cell clusters. Supplemental Table S1. Summary for each cell (barcode, sample group, cell type, and doublet result) for each cell in the snRNA-seq data. Supplemental Table S2. List of genes and gene ontologies that were up- and down-regulated in each cardiomyocyte cluster (CM1–CM6). Sub-tables include: Cluster CM1: upregulated genes (2.1), upregulated gene ontologies (2.2), downregulated genes (2.3), and downregulated gene ontologies (2.4)—Cluster CM2: upregulated genes (2.5), upregulated gene ontologies (2.6), downregulated genes (2.7), and downregulated gene ontologies (2.8)—Cluster CM3: upregulated genes (2.9), upregulated gene ontologies (2.10), downregulated genes (2.11), and downregulated gene ontologies (2.12)—Cluster CM4: upregulated genes (2.13), upregulated gene ontologies (2.14), downregulated genes (2.15), and downregulated gene ontologies (2.16)—Cluster CM5: upregulated genes (2.17), upregulated gene ontologies (2.18), downregulated genes (2.19), and downregulated gene ontologies (2.20)—Cluster CM6: upregulated genes (2.21), upregulated gene ontologies (2.22), downregulated genes (2.23), and downregulated gene ontologies (2.24) Supplemental Table S3. List of genes and gene ontologies that were up- and down-regulated in each fibroblast cluster (FB1–FB4). Sub-tables include:—Cluster FB1: upregulated genes (3.1), upregulated gene ontologies (3.2), downregulated genes (3.3), and downregulated gene ontologies (3.4)—Cluster FB2: upregulated genes (3.5), upregulated gene ontologies (3.6), downregulated genes (3.7), and downregulated gene ontologies (3.8)—Cluster FB3: upregulated genes (3.9), upregulated gene ontologies (3.10), downregulated genes (3.11), and downregulated gene ontologies (3.12)—Cluster FB4: upregulated genes (3.13), upregulated gene ontologies (3.14), downregulated genes (3.15), and downregulated gene ontologies (3.16) Supplemental Table S4. List of genes and gene ontologies that were up- and down-regulated in each smooth muscle cell cluster (SMC1-SMC6). Sub-tables include:—Cluster SMC1: upregulated genes (4.1), upregulated gene ontologies (4.2), downregulated genes (4.3), and downregulated gene ontologies (4.4)—Cluster SMC2: upregulated genes (4.5), upregulated gene ontologies (4.6), downregulated genes (4.7), and downregulated gene ontologies (4.8)—Cluster SMC3: upregulated genes (4.9), upregulated gene ontologies (4.10), downregulated genes (4.11), and downregulated gene ontologies (4.12)—Cluster SMC4: upregulated genes (4.13), upregulated gene ontologies (4.14), downregulated genes (4.15), and downregulated gene ontologies (4.16)—Cluster SMC5: upregulated genes (4.17), upregulated gene ontologies (4.18), downregulated genes (4.19), and downregulated gene ontologies (4.20)—Cluster SMC6: upregulated genes (4.21), upregulated gene ontologies (4.22), downregulated genes (4.23), and downregulated gene ontologies (4.24) Supplemental Table S5. List of genes and gene ontologies that were up- and down-regulated in each endothelial cell cluster (EC1–EC5). Sub-tables include:—Cluster EC1: upregulated genes (5.1), upregulated gene ontologies (5.2), downregulated genes (5.3), and downregulated gene ontologies (5.4)—Cluster EC2: upregulated genes (5.5), upregulated gene ontologies (5.6), downregulated genes (5.7), and downregulated gene ontologies (5.8)—Cluster EC3: upregulated genes (5.9), upregulated gene ontologies (5.10), downregulated genes (5.11), and downregulated gene ontologies (5.12)—Cluster EC4: upregulated genes (5.13), upregulated gene ontologies (5.14), downregulated genes (5.15), and downregulated gene ontologies (5.16)—Cluster EC5: upregulated genes (5.17), upregulated gene ontologies (5.18), downregulated genes (5.19), and downregulated gene ontologies (5.20) Supplemental Table S6. List of genes and gene ontologies that were up- and down-regulated in each immune cell cluster (Mph1-Mph4, Tcell1, and Tcell2). Sub-tables include:—Cluster Mph1: upregulated genes (6.1), upregulated gene ontologies (6.2), downregulated genes (6.3), and downregulated gene ontologies (6.4)—Cluster Mph2: upregulated genes (6.5), upregulated gene ontologies (6.6), downregulated genes (6.7), and downregulated gene ontologies (6.8)—Cluster Mph3: upregulated genes (6.9), upregulated gene ontologies (6.10), downregulated genes (6.11), and downregulated gene ontologies (6.12)—Cluster Mph4: upregulated genes (6.13), upregulated gene ontologies (6.14), downregulated genes (6.15), and downregulated gene ontologies (6.16)—Cluster Tcell1: upregulated genes (6.17), upregulated gene ontologies (6.18), downregulated genes (6.19), and downregulated gene ontologies (6.20)—Cluster Tcell2: upregulated genes (6.21), upregulated gene ontologies (6.22), downregulated genes (6.23), and downregulated gene ontologies (6.24) Supplemental Table S7. CellChat analysis of ligand-receptor communication from CM1 to other clusters.
