# Supplementary material for: Single-Nuclei RNA Sequencing Data Analysis: Cardiomyocyte-Specific Modified-mNA Targeting Cyclin D2 Turns Subpopulations of Adult Pig Cardiomyocytes, Fibroblasts, and Smooth Muscle Cells Back to Neonatal Characteristics at Day 2 After SMRT Intervention
Source: Biomolecules. 2026 Oct 10;16(10):1477. doi: 10.3390/biom16101477 (PMC13702326; doi:10.3390/biom16101477)
Supplement: Supplementary file 1 [file biomolecules-16-01477-s001.zip › Proof read supplemental figures.pdf]

Supplementary Figures

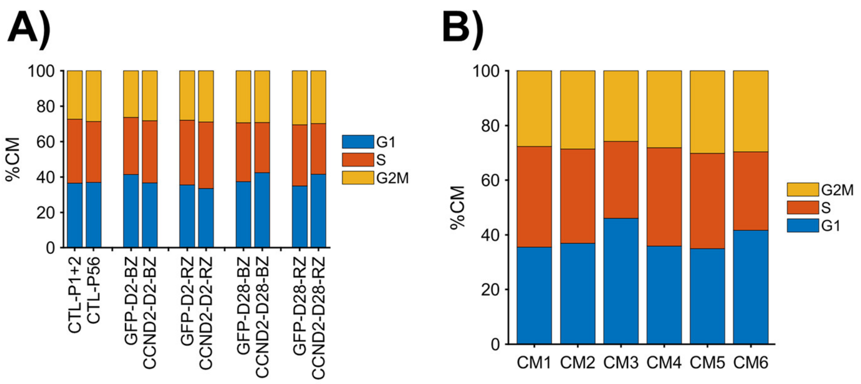

Supplementary Figure S1. Seurat's cell cycle scoring analysis of cardiomyocyte. (**A**) Stacked bar plot showed the distribution of cardiomyocytes at the G1, S, and G2M phase; each column corresponds to a sample group. (**B**) The same stacked bar plot to (A), but each column corresponds to a cardiomyocyte cluster.

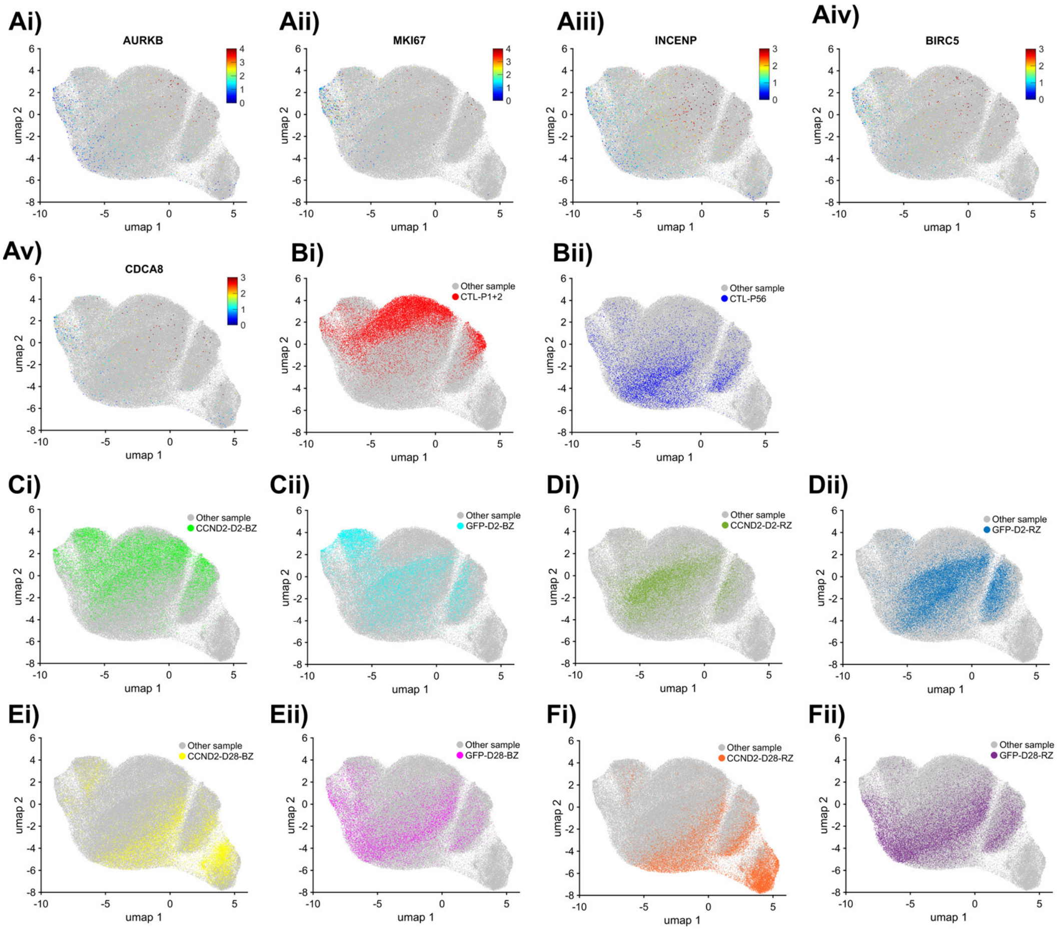

Supplementary Figure S2. Cell cycle-specific Autoencoder analysis of cardiomyocyte. (**A**) Distribution of cells expressing five proliferation markers *AURKB* (**Ai**), *MKI67* (**Aii**), *INCENP* (**Aiii**), *BIRC5* (**Aiv**), and *CDCA8* (**Av**); each dot represents a cell, the dot color reflects the expression of each marker, and the gray cell did not express the marker; we did not detect any area in which all markers were enriched. (**B-E**) Distribution of cardiomyocytes in specific heart groups, in pair: (**B**) neonatal P1+2 (**Bi**) vs adult P56 (**Bii**); (**C**) CCND2-D2-BZ (**Ci**) vs GFP-D2-BZ (**Cii**); (**D**) CCND2-D2-RZ (**Di**) vs GFP-D2-RZ (**Dii**); (**E**) CCND2-D28-BZ (**Ei**) vs GFP-D28-BZ (**Eii**); (**F**) CCND2-D28-RZ (**Fi**) vs GFP-D28-RZ (**Fii**).

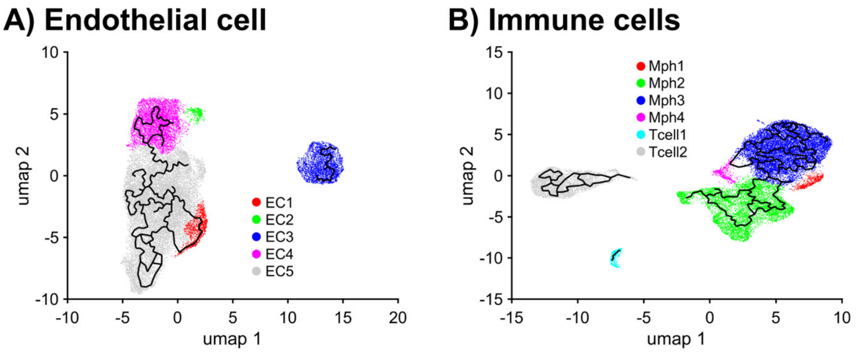

Supplementary Figure S3. Trajectory analysis of endothelial cells (A) and immune cell clusters (B).
